# Heparin-gold nanoparticles for enhanced microdialysis sampling

**DOI:** 10.1007/s00216-017-0447-y

**Published:** 2017-06-29

**Authors:** Susan Giorgi-Coll, Holly Blunt-Foley, Peter J. Hutchinson, Keri L.H. Carpenter

**Affiliations:** 1 Division of Neurosurgery, Department of Clinical Neurosciences, University of Cambridge, Box 167, Cambridge Biomedical Campus, Cambridge, CB2 0QQ UK; 2 Wolfson Brain Imaging Centre, Department of Clinical Neurosciences, University of Cambridge, Box 65, Cambridge Biomedical Campus, Cambridge, CB2 0QQ UK

**Keywords:** Microdialysis, Cytokines, Heparin, Gold nanoparticles, Brain injury

## Abstract

Cerebral microdialysis is a sampling technique which offers much potential for understanding inflammatory pathophysiology following traumatic brain injury (TBI). At present, the recovery of cytokines via microdialysis in clinical studies is not straightforward primarily due to their size, steric properties and low concentrations. Heparin and heparin-coated microspheres have previously shown promise as cytokine-binding agents for enhanced microdialysis sampling in animal models (Duo and Stenken in Anal Bioanal Chem 399(2):773–82, 2011; Anal Bioanal Chem 399(2):783–93, 2011). However, there are several factors limiting application for microdialysis in patients. The aim of this study was to produce heparin-coated gold nanoparticles as cytokine capture agents for enhanced microdialysis sampling, potentially applicable to a clinical setting. Gold nanoparticles (AuNP) were chemically conjugated to heparin via a bifunctional polyethylene glycol (PEG) linker. The heparin-AuNP (AuNP-Hep) were characterised, demonstrating the successful addition of heparin to the gold surface. The performance of the AuNP-Hep during in vitro testing was compared both to current methodology (Helmy et al. in J Neurotrauma 26(4):549–61, 2009) and to the heparin-coated microspheres developed by Duo and Stenken (Anal Bioanal Chem 399(2):773–82, 2011; Anal Bioanal Chem 399(2):783–93, 2011). The AuNP-Hep yielded a higher recovery of cytokines compared to current methodology and heparin-coated microspheres, during in vitro testing designed to mimic the human environment and the intensive care unit. In this study, AuNP-Hep were developed for enhanced microdialysis sampling of cytokines, potentially applicable in a clinical setting. Based on the success of the AuNP-Hep in vitro, the proposed method offers an alternative to the use of current protocols that rely on a blood product (albumin) for microdialysis sampling of cytokines in patients.

**Electronic supplementary material:**

The online version of this article (doi:10.1007/s00216-017-0447-y) contains supplementary material, which is available to authorized users.

## Introduction

Cerebral microdialysis is a monitoring technique used in traumatic brain injury (TBI) patients which has both clinical and research applications. Microdialysis enables sampling from the extracellular space in vivo, thereby providing a unique opportunity to study underlying inflammatory processes that occur in the brain after traumatic injury, as well as potentially identifying therapeutic targets. Microdialysis sampling is based on the free diffusion of analytes across a semi-permeable membrane with a nominal molecular weight cut-off (MWCO). The membrane is attached to the inlet and outlet tubing through which perfusion fluid (PF) is slowly passed and collected [1]. Cytokines are key mediators in the inflammatory processes which can occur following TBI [2, 3]. Cerebral microdialysis has previously been applied for the sampling of cytokines from TBI patients 2–8 days after injury [4, 5].

**Fig. 1:**
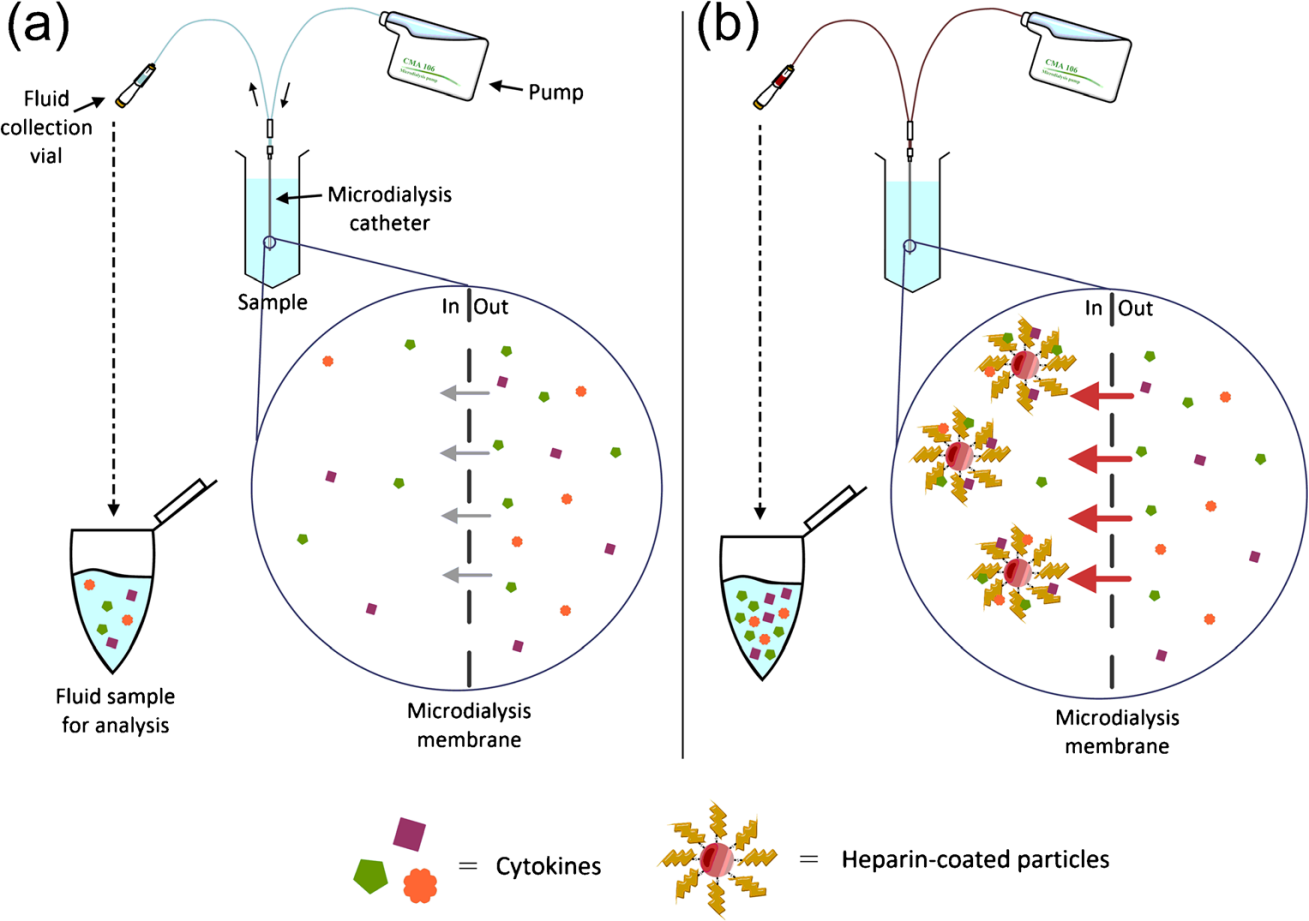
A schematic overview demonstrating the recovery of cytokines in vitro, by **a** standard microdialysis, and **b** enhanced microdialysis using heparin-coated particles

The recovery of proteins such as cytokines via microdialysis sampling poses a significant challenge. Proteins are difficult to collect due to their low diffusion coefficients across the microdialysis membrane and their typically low concentrations (pg/mL) in the brain [1]. PF containing affinity-based trapping agents targeted to the protein of interest has been applied to overcome this problem [1]. By binding the protein within the microdialysis catheter, the trapping agent produces an artificially low concentration in the PF, thereby driving diffusion across the membrane [6]. Increased diffusion via mass transport, together with a chemical reaction across the membrane, causes more of the protein analyte to enter the catheter and thereby improves recovery (Fig. 1). At present, PF supplemented with human serum albumin (HSA) is the gold standard for improving cytokine recovery during clinical microdialysis [7]. However, the acquisition of blood products such as HSA for clinical use is becoming increasingly difficult in Europe due to licencing changes in pharmacy manufacturing units. It is imperative for the continued clinical use of microdialysis that alternative strategies for improving cytokine recovery which do not require blood products, such as those using affinity-based trapping agents, are developed.

Heparin has been applied as one such cytokine capture agent during in vitro microdialysis [6]. Heparin, a highly polar and linear polysaccharide belonging to a family of glycosaminoglycan (GAG), is known to bind a range of cytokines and chemokines with micromolar to nanomolar affinity [1]. The main advantage of using heparin over antibodies as a capture agent is the faster dissociation rate associated with heparin-cytokine interactions, which allows cytokines to be removed after sampling for detection. Furthermore, heparin is inexpensive, widely available and chemically more stable than antibodies [8]. Wang and Stenken reported a 2–5-fold increase in the relative recovery (RR) of five cytokines, namely IL-4, IL-6, IL-7, MCP-1 and TNF-α, using microdialysis perfusion fluid supplemented with 0.1 μM heparin [6]. At this concentration, a low loss of heparin across the 100-kDa MWCO microdialysis membrane (2.7 ± 0.9%, *n* = 3) was reported [6]. This is thought to be due to its rigid linear polymeric structure and large hydrodynamic radius, both of which limit diffusion across the catheter membrane.

Due to safety concerns regarding free heparin, Stenken and colleagues also developed and applied heparin-coated microspheres (Hep-beads) for enhancing cytokine recovery during microdialysis [1, 9]. In vitro studies using the Hep-beads showed that the concentration of Hep-beads did not change during sampling, suggesting that no Hep-beads were able to pass through the microdialysis membrane [8]. In vivo results in rats showed that the same concentration of heparin elicited the same improvement of RR, whether free or immobilised onto a bead surface, thereby demonstrating the potential of Hep-beads as a viable alternative to free heparin [8]. However, the size of the beads means that they are liable to settle in the delivery syringe during microdialysis sampling and therefore require a rotating platform in order to work, which is not practical in a clinical setting.

In this study, AuNP were selected as an alternative particle for their biocompatibility, stability in aqueous solutions, unique optical properties and low cost. The small size of AuNP enables them to remain in suspension after functionalisation, and therefore, they would not require rotation during microdialysis sampling as is necessary with the Hep-beads. In addition, the unique surface plasmon resonance of AuNP provides opportunities for the future development of optical tests for cytokines of interest. Heparin-functionalised gold nanoparticles (AuNP-Hep) were synthesised, characterised and tested in vitro for their capability to enhance recovery of cytokines during microdialysis in a clinical setting. Heparin-coated microspheres were also prepared (as described by Duo and Stenken [8, 9]) and tested in vitro. The two heparin-particle types were compared to the current method (PF supplemented with HSA) in an in vitro setting that closely approximates the clinical environment, to ascertain whether either will be suitable for use in human patients. As yet, no study has tested heparin-particles in this context, and it is a necessary step before implementing it in human patients.

## Materials and methods

### Materials

All reagents were of analytical grade, purchased from Sigma-Aldrich (Poole, Dorset, UK) and used as received unless otherwise stated. Sodium phosphate dibasic, sodium chloride, sodium dodecyl sulphate (SDS) and potassium chloride were purchased from BDH laboratory supplies (Poole, UK). Millipore Amicon ultra 0.5 mL centrifuge filter tubes (10,000 and 100,000 MWCO) were purchased from Thermo Fisher (Loughborough, UK). Bangs Laboratories polybead amino microspheres (6.0 μm o.d., 8.26 × 10^7^ beads/mL in deionised water containing 0.1% SDS) were purchased from Polysciences Europe GmbH (Eppelheim, Germany). Kova Glasstic microscope slides (10 grids) were purchased from VWR (Lutterworth, UK). Holey carbon film 300 mesh copper grids were purchased from Agar Scientific (Stansted, UK). CMA71 brain microdialysis catheters (100 kDa nominal MWCO, 10 mm polyarylethersulfone [PAES] membrane length), microdialysis vials, CMA106 microdialysis pumps and corresponding batteries and syringes were purchased from M Dialysis AB (Stockholm, Sweden). ProcartaPlex human cytokine and chemokine (panel 1A) 34-plex bead assays and human cytokine and chemokine standard mixes A and B were purchased from Affymetrix eBioscience (Hatfield, UK).

All centrifugation steps were performed using a Hettich Universal 320R centrifuge. In vitro sampling with heparin beads was performed using a Grant-Bio PS-3D Sunflower mini-shaker. Citrate AuNP synthesis and in vitro microdialysis sampling with AuNP-heparin were performed using a VWR advanced hotplate magnetic stirrer with temperature probe. Catheters were held in place during in vitro sampling using a triple lumen cranial access device (Technicam, Newton Abbott, UK). UV-Vis absorption spectra (200–700 nm) were recorded on a Hitachi U-3000 spectrophotometer at room temperature. Quartz cuvettes with a 1-cm path length were used. Azure A assay absorbance intensity readings were recorded using an Anthos Multiread 400 plate reader. Transmission electron microscope (TEM) images were obtained using a FEI Tecnai F20 FEGTEM operating at 200 kV. Luminex multiplex assays were analysed using a Luminex 200 analyser (Luminex Corporation, Austin, TX, USA) operating with Luminex xPonent software. Wash steps were performed using either a BioTek ELx405 automated microplate washer (Winooski, VT, USA) or a ProcartaPlex handheld magnetic plate holder.

### Buffers and solutions

All high-purity deionised water (dH_2_O) used was of HPLC grade (18.2 MΩ cm, Millipore Direct Q5 UV water purification system with LC-Pak polisher). Microdialysis PF was made in-house (147 mM NaCl, 2.7 mM KCl, 1.2 mM CaCl_2_, 0.85 mM MgCl_2_; pH ∼ 6.0), to the same specifications as the manufactured PF used for brain microdialysis in patients in our neurocritical care unit (NCCU). 2-(*N*-morpholino)methanesulfonic acid buffer (MES buffer) was made at 50 mM, pH 5.5 with 0.01% (*w*/*v*) SDS. HEPES buffer was made at 50 mM, with 100 mM NaCl (pH 7.4). Phosphate-buffered saline (PBS) was made to the concentrations and pH values specified.

### AuNP synthesis and characterisation

Sixteen-nanometer citrate-stabilised gold nanoparticles (cAuNP) were synthesised according to the method of Turkevich et al. [10]. The concentration of AuNP was calculated based on the extinction at 520 nm and the extinction coefficient 2.4 × 10^8^ M^−1^ cm^−1^ [11]. TEM was used to characterise the size and morphology of the AuNP; 10 μL of the AuNP in solution was placed onto a holey carbon film 300 mesh copper grid. Excess solvent was removed from the sample by touching the side of the grid with adsorbent tissue paper. The grid was dried overnight at room temperature before analysis by TEM, as previously described. Measurement of the cAuNP was performed using ImageJ software.

### Preparation of the heparin-AuNP

Heparin-AuNP were prepared using a bifunctional PEG linker, with both a thiol functional group for tethering onto the surface of the AuNP, and a terminal amine which can be coupled onto the heparin. The PEG linker was used to increase the size of the AuNP, as well as to impart additional stability under biological conditions [12, 13]. The PEG was self-assembled onto the AuNP as follows: a *w*/*v* ratio of 1 PEG:1 AuNP was self-assembled by adding 5 mg of HS-poly(ethylene glycol)-NH_2_ (average MW 2000; PEG) to 5 mL of cAuNP, prepared as described above. The mixture was gently stirred for 12 h at room temperature. Unbound PEG was removed by centrifuge filtration (100,000 MWCO; 15 min, 8000×g), after which the AuNP-PEG were resuspended in 2.5 mL MES buffer with SDS. Coupling of the heparin to the AuNP-PEG was performed using sulfo-NHS/EDC chemistry, as previously applied by Benoit et al., among others [14, 15]; 25 μL of an EDC/sulfo-NHS solution (0.1 M EDC and 0.1 M sulfo-NHS in dH_2_O) was added to 100 μL of heparin (10 mg/mL in MES buffer with SDS). The solution was reacted for 15 min at room temperature with gentle stirring, after which 2.5 mL of the AuNP-PEG (prepared as above) was added. The solution was vortex mixed before being gently stirred for 2 h at room temperature. After incubation, the AuNP-heparin (AuNP-Hep) were washed successively with MES buffer with SDS and 100 mM ethanolamine, to quench unreacted carboxyl groups; MES buffer with SDS; and finally PF with 0.05% (*w*/*v*) sodium azide and 1 mg/mL human serum albumin (HSA). Washing was performed by centrifuge filtration (100,000 MWCO; 15 min, 8000×g). After the final wash, the AuNP-Hep were resuspended in PF with 0.05% (*w*/*v*) sodium azide and 1 mg/mL HSA. The AuNP-Hep were stored at 4 °C.

Once purified, modification of the AuNP surface was determined by UV-Vis spectrophotometry at ca. 520 nm. The amount of immobilised heparin was detected using the heparin dye Azure A [16], as per the method of Bai et al. [17]. A standard curve comprising heparin concentrations between 0.195 and 50 μM was prepared in HEPES buffer. For the assay, 180 μL of Azure A dye (150 μM, in HEPES buffer) was mixed with 20 μL of standard or sample in a 96-well microplate, before being incubated for 30 min at room temperature in the dark. The absorption of the samples was then measured by a plate reader at 620 nm.

### Cytokine binding

The cytokine-binding capability of the AuNP-Hep was determined by incubation in PF containing 34 human cytokines; AuNP-PEG treated in the same way were used as a negative control. Unbound cytokines were removed by washing. Bound cytokines were removed from the heparin using a dissociation step in 20% (*v*/*v*) acetonitrile as described by Duo and Stenken [8]. The concentration of cytokine bound to the AuNP-Hep was then determined by multiplex immunoassay and compared with the control. The storage buffer was first removed from the AuNP-PEG and AuNP-Hep (ca. 3.5 nM; 1 mL of each) by centrifuge filtration (100,000 MWCO; 15 min, 8000×g). The AuNP were then resuspended in an external solution (ES) comprising PF with 0.05% (*w*/*v*) sodium azide, 1 mg/mL HSA and 34 human cytokines, prepared as follows. The cytokine standards, received as a set of two lyophilised powders (A and B) containing a mixture of different cytokines, were resuspended in accordance with the manufacturers’ instructions and subsequently diluted to 1:100 in PF with 0.05% (*w*/*v*) sodium azide and 1 mg/mL HSA. Following this dilution, the final cytokine concentrations used in the experiments are as listed in Table 1. A 2-h incubation was performed at room temperature with gentle shaking (120 rpm). Unbound cytokines were removed by washing three times with PF with 0.05% (*w*/*v*) sodium azide and 1 mg/mL HSA; wash steps were performed by centrifuge filtration (100,000 MWCO; 15 min, 8000×g). A fourth and final wash was performed in PF, after which the cytokines were removed from the surface of the Hep-beads and AuNP-Hep using a dissociation step modified from Duo and Stenken [9]. The AuNP-PEG and AuNP-Hep were resuspended in PF containing 10% (*v*/*v*) acetonitrile and incubated for 12–14 h at 4 °C. After this dissociation step, the AuNP-PEG and AuNP-Hep were centrifuge filtered (100,000 MWCO; 15 min, 8000×g), and the supernatant containing the cytokines retained. Samples were stored at −75 °C for up to 2 months prior to analysis.

**Table 1:** Concentrations of cytokine standards used during cytokine binding and in vitro sampling experiments. Standards were diluted to concentrations that reflect those found in the brain’s extracellular fluid by Hutchinson et al. [3], assuming a 20% relative recovery (RR) [7]

Cytokine/chemokine	Concentration (pg/mL)
Eotaxin	25
GM-CSF	543
GRO-alpha	95
IFN-alpha	22.5
IFN-gamma	306
IL-1alpha	22
IL-1beta	84
IL-1ra	1438
IL-2	175
IL-4	463
IL-5	263
IL-6	334
IL-7	20
IL-8	89
IL-9	315
IL-10	92
IL-12p70	262
IL-13	88
IL-15	125
IL-17alpha	74
IL-18	280
IL-21	319
IL-22	1082
IL-23	559
IL-27	1002
IL-31	546
IP-10	88
MCP-1	65
MIP-1alpha	64
MIP-1beta	135
RANTES	36
SDF-1alpha	398
TNF-alpha	267
TNF-beta	238

### Preparation of the heparin beads

Conjugation of heparin onto Bangs’ amine-functionalised polymer microspheres was achieved using a modified version of the method described by Duo and Stenken [8, 9]. The polymer beads (100 μL of stock suspension, 8.26 × 10^7^ beads/mL) were washed three times with 400 μL of PBS (0.2 M, pH 7.0) by centrifuge filtration (10,000 MWCO; 10 min, 14,000×g). Following centrifugation, the microspheres were resuspended in 500 μL of 6 mg/mL solution of heparin in PBS (0.2 M, pH 7.0). The beads were diluted to 1:2 in 6 mg/mL sodium cyanoborohydride, and the solution was incubated for 48 h on a microplate shaker (1000 rpm, at room temperature). After incubation, the microspheres were washed three times with 400 μL of sodium acetate buffer (0.2 M, pH 7.0) by centrifuge filtration (100,000 MWCO; 10 min, 14,000×g). After washing, the microspheres were resuspended in 1 mL sodium acetate buffer (0.2 M, pH 7.0) containing 1 mg/mL SDS; 0.5 mL acetic anhydride was added dropwise to the suspension, and the solution was incubated at room temperature for 1 h to deactivate any unbound amine groups. After incubation, the microspheres were washed successively with 300 μL sodium acetate buffer (0.2 M, pH 7.0) with 1 mg/mL SDS, dH_2_O, and finally in 400 μL of 10 mM PBS (pH 7.4) with 0.05% (*w*/*v*) sodium azide. The microspheres were then resuspended in 100 μL of PBS (10 mM, pH 7.4) with 0.05% (*w*/*v*) sodium azide and counted using a haemocytometer. The heparin beads were stored at 4 °C.

### In vitro microdialysis sampling

Prior to microdialysis sampling, 1.5 mL of AuNP-PEG was washed by centrifuge filtration (100,000 MWCO; 15 min, 8000×g) to remove the buffer and resuspended in 1.5 mL of PF with 0.05% (*w*/*v*) sodium azide and 1 mg/mL HSA. The AuNP-Hep were prepared as previously described. The Hep-beads were prepared as follows: a 4 × 10^6^ beads/mL concentration was achieved by diluting 176.5 μL of the beads (as calculated for the final 3 mL volume) in 823.5 μL of PF. The beads were divided into two equal parts, washed by centrifuge filtration (10,000 MWCO; 10 min, 14,000×g) and then resuspended in 1.5 mL of PF with 0.05% (*w*/*v*) sodium azide and either 35 or 1 mg/mL HSA. The solutions were then loaded into syringes and CMA107 microdialysis perfusion pumps.

**Fig. 2:**
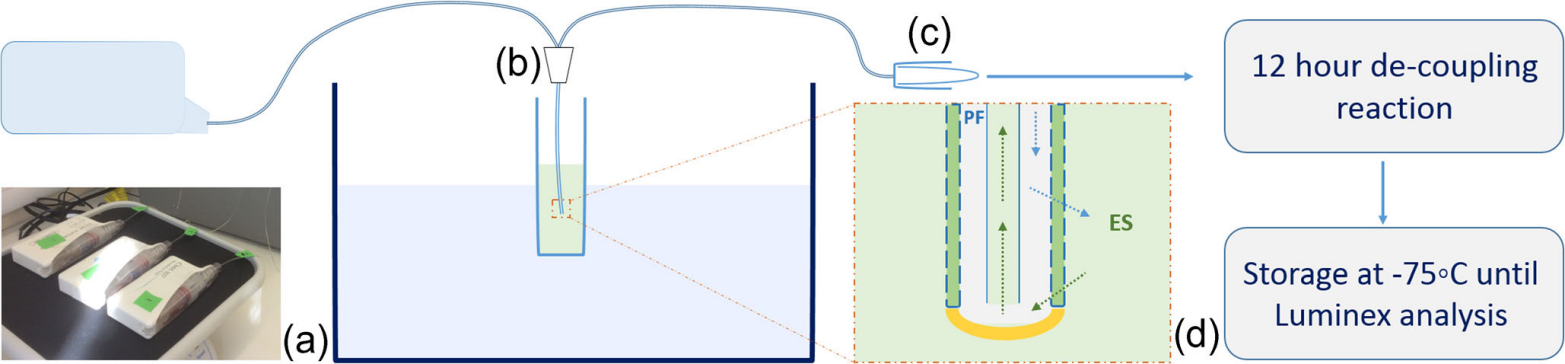
Diagram of the experimental setup. **a** Three CMA microdialysis pumps with various perfusion fluids (as described in Table 2). **b** Catheter setup via triple bolt to sample external solution (ES) in 37 °C water/glycerol bath. **c** Collection vial. **d** Magnified schematic of microdialysis catheter tip, demonstrating direction of perfusate flow and diffusion across the outer catheter membrane

In vitro microdialysis sampling was performed in an ES containing 34 human cytokines. The ES was prepared as previously described for the cytokine-binding experiments; 25 mL of ES was made up in a 50-mL centrifuge tube. The tube was suspended using a clamp stand in either a thermostatically controlled water bath (for the Hep-bead sampling) or glycerol bath (for the AuNP-Hep sampling) set to 37 °C. Very gentle agitation of the external solution was applied using a magnetic stirrer. Two or three CMA71 brain microdialysis catheters were placed into the external solution through a triple bolt cranial access device, which was secured within the centrifuge tube using self-adhesive plastic film. Each catheter was perfused at 0.3 μL/min using CMA107 pumps, with syringes loaded with ca. 1.5 mL of PF, prepared as described above. The different types of PF used in this study are summarised in Table 2. Once connected, pumps were placed onto a rotating shaker platform set at low speed. The microdialysate samples were collected in microdialysis vials at the end of each catheter. Pumps and collection vials were kept at the same height on either side of the water/glycerol bath, to nullify any hydrostatic pressure differences. Sampling was performed for 48 h in total; the microdialysis vials were changed every 12 h. Samples were stored at 4 °C until the dissociation step was carried out (within 24 h of sampling). A schematic of the in vitro sampling setup is shown in Fig. 2. After sampling, the microdialysate samples were diluted to a 1:2 concentration in PF supplemented with 20% (*v*/*v*) acetonitrile and incubated at 4 °C for 10–12 h to remove the cytokines from the surface of the Hep-beads and AuNP-Hep. After the dissociation step, the AuNP-PEG and AuNP-Hep were removed by centrifuge filtration (100,000 MWCO; 15 min, 8000×g), and the supernatant containing the cytokines retained. Samples were stored at −75 °C for up to 1 month prior to analysis.

**Table 2:** A summary of the different types of PF compared during this study. The estimated average perfusion fluid concentrations of Hep-beads and AuNP-Hep used were 44.0 × 10^5^ beads/mL and 3.5 nM, respectively

	Pump 1	Pump 2	Pump 3
Hep-beads			
Repeat 1	PF with 1 mg/mL HSA	Hep-beads in PF with 35 mg/mL HSA	Hep-beads in PF with 1 mg/mL HSA
Repeat 2	PF with 1 mg/mL HSA	Hep-beads in PF with 35 mg/mL HSA	Hep-beads in PF with 1 mg/mL HSA
AuNP-Hep			
Repeat 1	PF with 1 mg/mL HSA	AuNP-Hep in PF with 1 mg/mL HSA	AuNP-PEG in PF with 1 mg/mL HSA
Repeat 2	PF with 1 mg/mL HSA	AuNP-Hep in PF with 1 mg/mL HSA	–

### Sample analysis

Quantitative analysis of cytokine concentration was performed using an eBiosciences ProcartaPlex 34-plex human cytokine immunoassay kit. The samples were thawed and gently vortex mixed before analysing. Twenty-five microliters of the sample was used per well; all samples were analysed in duplicate. The assay was performed as per the manufacturers’ instructions. Wash steps were carried out using either an automated plate washer (for the Hep-bead analysis) or a handheld magnetic plate holder. All assays were analysed on a Luminex 200 platform. The RR was determined based on the average of the duplicate measurements for each sample. Data analysis was performed using xPonent and Origin software.

## Results and discussion

### AuNP synthesis and characterisation

**Fig. 3:**
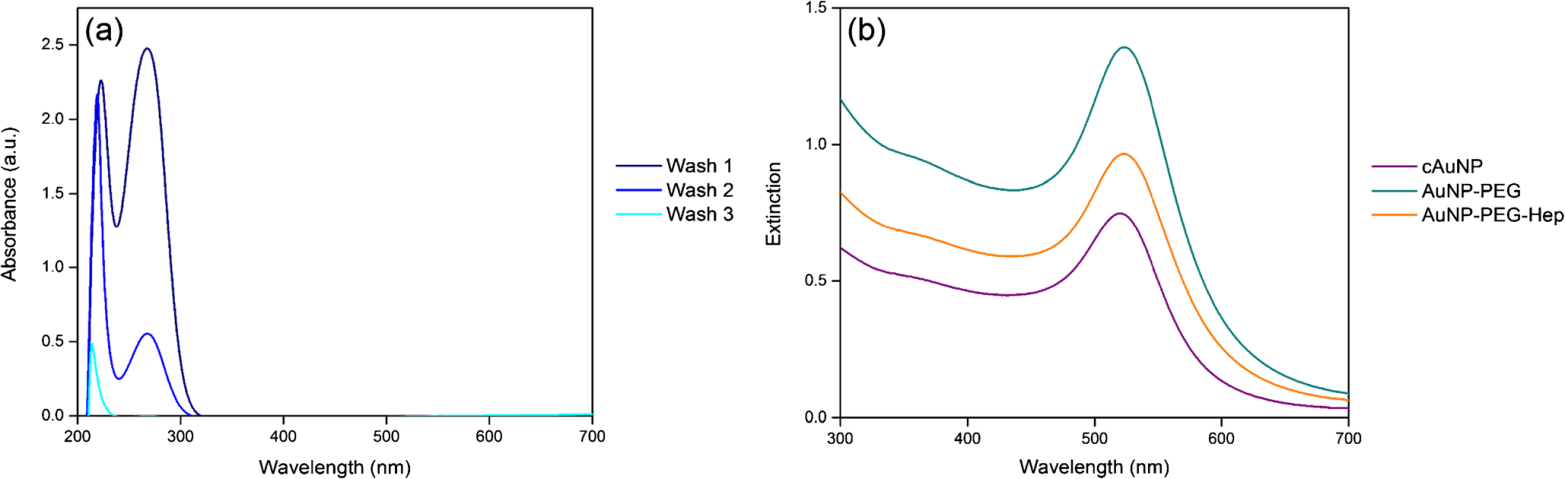
UV-Vis absorption and extinction spectra during AuNP-Hep synthesis. **a** Removal of unbound heparin from the AuNP, as indicated by the decreasing absorption at approximately 275 nm (*A*_max_ heparin = ca. 275 nm) during washing. **b** Extinction spectra of cAuNP, showing the *A*_max_ at ca. 520 nm. After the addition of PEG and then heparin to the AuNP surface, the peak extinction increases by 1–2 nm per surface modification. The AuNP-PEG were concentrated by 2× after the addition of the PEG linker

AuNP were synthesised according to the citrate reduction method of Turkevich et al., as described in the “Materials and methods” section [18]. Heparin was added to the surface of the AuNP via a two-step procedure, in which a PEG linker was first bound to the AuNP followed by the subsequent addition of the heparin. Unbound heparin was removed by three wash steps, the supernatant of which was analysed by UV-Vis spectrophotometry (Fig. 3a). Removal of the unbound heparin is shown by the decreasing peak at its absorbance maximum at ca. 275 nm [19]. This result was verified by additional analysis of the wash supernatants by Azure A assay. The results showed decreasing concentrations of heparin after each wash step, with no heparin detected in the final wash, in agreement with the UV-Vis results (data not shown). cAuNP absorb light in the visible spectrum with a maximum at ca. 520 nm, yielding an extinction spectrum as shown in Fig. 3b. The concentration of the newly synthesised cAuNP shown in Fig. 3b was determined to be 3.37 nM using the extinction coefficient 2.4 × 10^8^ M^−1^ cm^−1^ [11, 20–22]. The binding of PEG and subsequently the heparin to the surface of the AuNP is demonstrated by an increase in peak extinction of approximately 1–2 nm per addition, as shown in Fig. 3b. This shift to the red region of the spectrum is indicative of modification of the surface of the AuNP, resulting in a size increase which is reflected by increased absorption. The results shown confirm the successful addition of PEG and heparin to the particle surface.

**Fig. 4:**
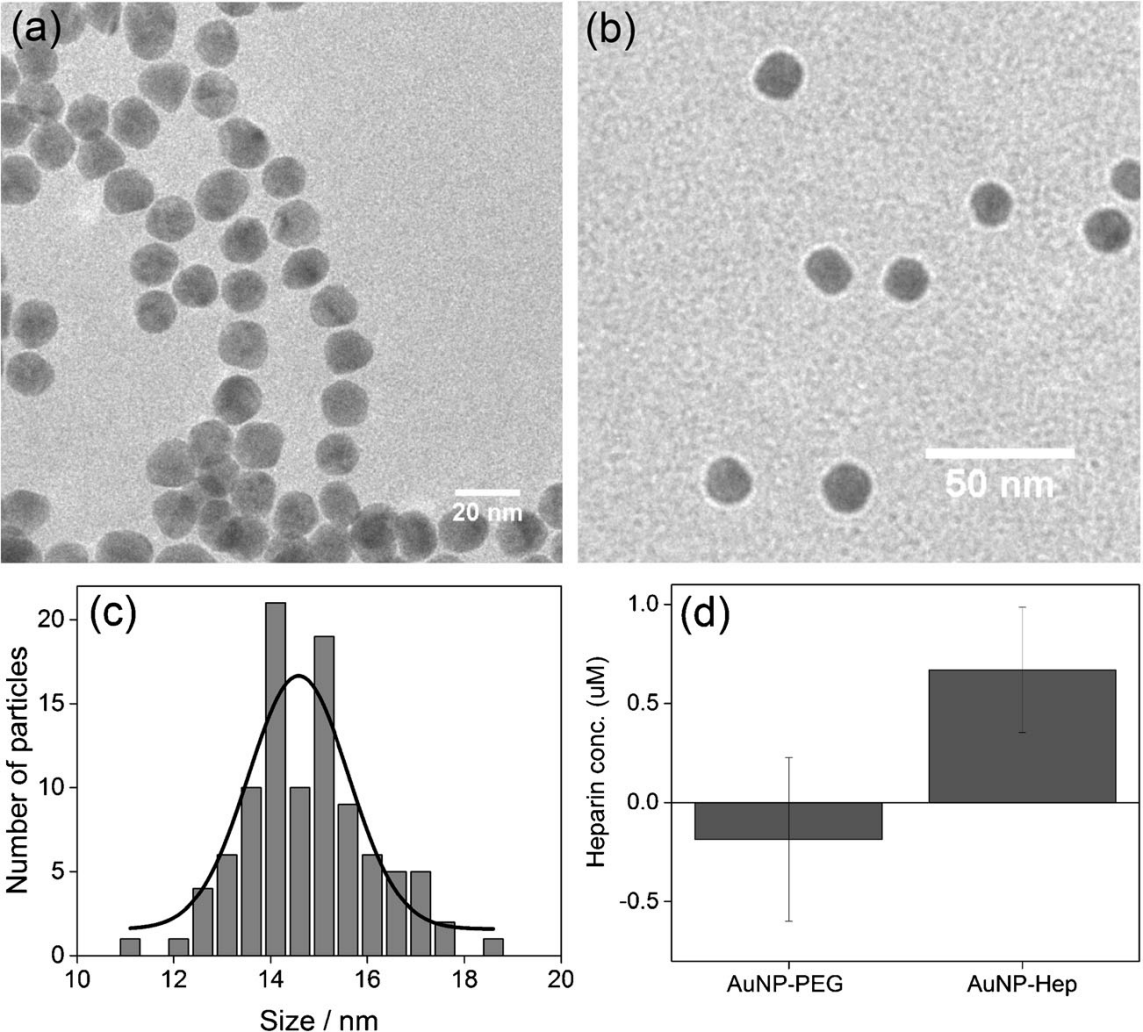
Characterisation of the AuNP-Hep by TEM and Azure A assay. TEM images of the citrate AuNP and the AuNP-Hep are shown in **a** and **b**, respectively. **c** The average size of the AuNP core, estimated from the TEM images, was 15.0 ± 1.3 nm (*n* = 100). **d** The concentration of heparin on the surface of the AuNP-Hep determined by Azure A assay. The average of three separate batches of AuNP-Hep, analysed in triplicate, is shown. AuNP-PEG were used as a negative control

TEM was used to visualise the cAuNP and AuNP-Hep (shown in panels a and b of Fig. 4, respectively). The average diameter of the cAuNP was measured from the TEM images; results are shown in Fig. 4c. The calculated average diameter of the cAuNP was 15.0 ± 1.3 nm (*n* = 100). The presence of heparin on the surface of the AuNP-Hep was confirmed by Azure A assay. The assay showed that heparin was present on the AuNP-Hep and absent on the AuNP-PEG negative control. A ratio of 108 heparin molecules per AuNP was estimated based on the quantity of heparin determined by Azure A assay.

### Cytokine binding

**Fig. 5:**
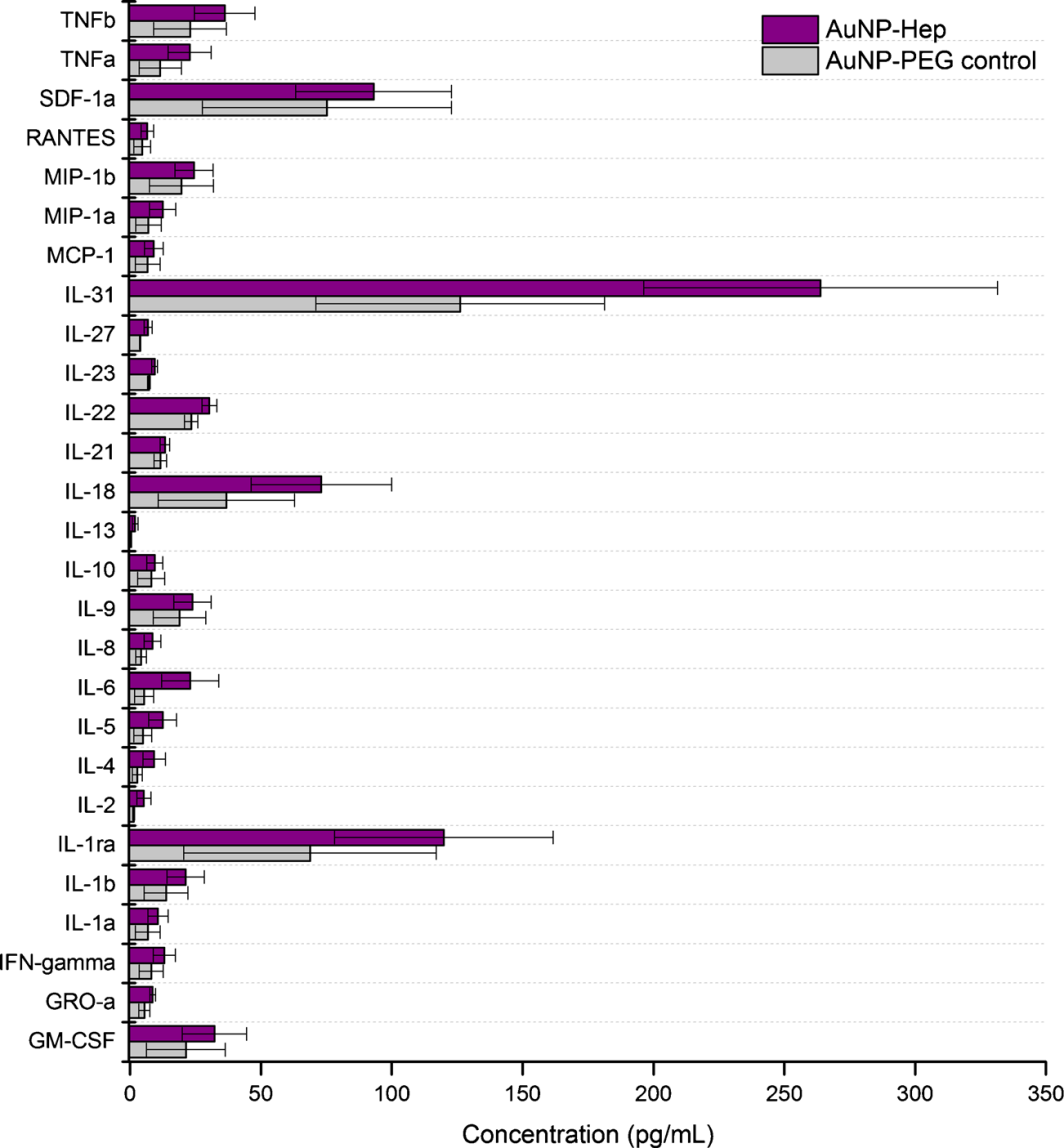
Results of the cytokine-binding test, wherein the AuNP-Hep were incubated in a mixed standard solution of the 34 human cytokines. Unbound cytokines were then removed by washing, followed by decoupling of bound cytokines and detection. The concentration of the cytokines bound to the AuNP-Hep, versus the concentration bound to the AuNP-PEG negative control, is shown. All cytokines with concentrations of >5 pg/mL are shown; the remaining data can be found in ESM Table S2. The data shown here represents the average of three repeat experiments, performed and analysed in duplicate (*error bars* = SEM). No significant difference was found between the AuNP-Hep and the AuNP-PEG control concentrations (*p* > 0.05; one-way ANOVA and paired sample *t* test)

The cytokine-binding capacity of the AuNP-Hep was determined by incubation in a solution containing 34 human cytokines, followed by four wash steps, decoupling and detection. Identical treatment of AuNP-PEG served as a negative control. Figure 5 shows the concentration of different cytokines obtained using the AuNP-Hep versus the control. Additional data showing the recovery of cytokines from the sampling solution, expressed as relative recovery (in %), is provided in the Electronic supplementary material (ESM) Table S1. Overall, application of the AuNP-Hep resulted in enhanced cytokine binding, as shown by the higher concentrations of all 34 cytokines decoupled from the AuNP-Hep when compared with the PEG control. However, no significant differences were seen in relative recovery when comparing the AuNP-Hep with the control. This was due to the presence of cytokines in the control samples, possibly due to steric properties in the PEG coating.

### In vitro microdialysis sampling

**Fig. 6:**
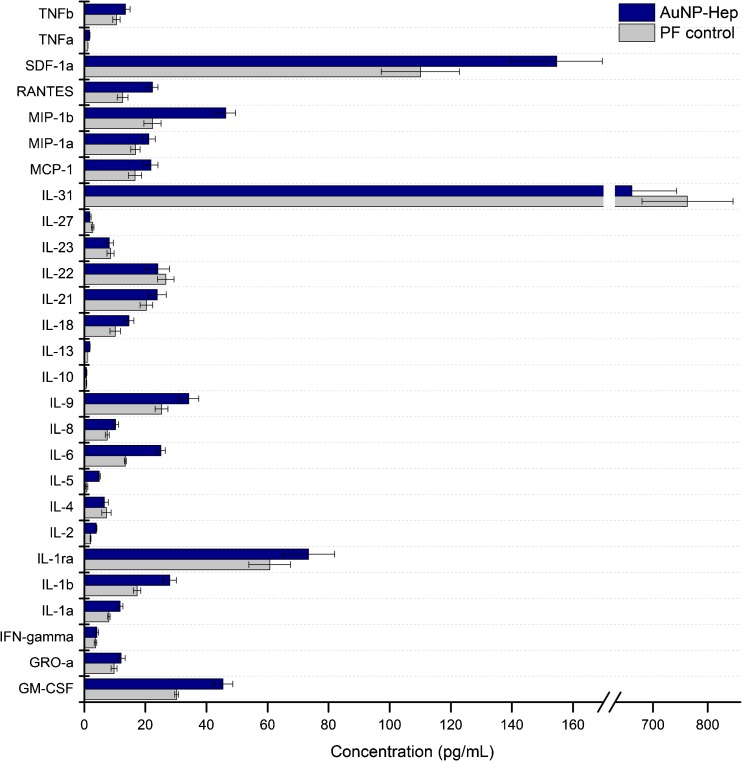
The concentrations of 34 human cytokines from in vitro microdialysis sampling using AuNP-Hep. Average concentrations from 48 h worth of sampling (at 12 h sample intervals), from two repeat experiments analysed in duplicate, are shown. All cytokines with concentrations of >5 pg/mL are shown; the remaining data can be found in ESM Table S3. *Error bars* denote SEM. No significant difference was found between the AuNP-Hep and the PF with HSA control concentrations (*p* > 0.05; one-way ANOVA and paired sample *t* test)

The cytokine-binding enhancement of the AuNP-Hep during microdialysis sampling was determined using an in vitro sampling setup designed to resemble microdialysis monitoring in TBI patients in our NCCU. The in vitro experiments were thus carried out using the same microdialysis catheters (100 K MWCO), pumps, flow rate (0.3 μL/min), collection vials and PF composition as used clinically. Sampling was performed at 37 °C, using three probes placed in a mixed standard solution of 34 human cytokines under gentle agitation, diluted to concentrations representative of those previously detected in brain extracellular fluid and assuming a 20% RR [3, 5]. The probes were perfused with different test solutions as detailed in the “Materials and methods” section. After sampling, a decoupling step was performed on all perfusates to remove any bound cytokines from the heparin on either the Hep-beads or the Hep-AuNP. The resulting cytokine concentrations sampled using the AuNP-Hep relative to the PF control are shown in Fig. 6. As demonstrated, sampling using AuNP-Hep resulted in a higher recovered concentration in the majority of the 34 cytokines tested for. Only the IL-31 concentration was lower when sampled by AuNP-Hep, although this varied between runs, and both the test and control solutions were at considerably higher concentration and recovery rate compared with the other cytokines in this study. The high concentration of IL-31 in the cytokine standard mix is thought to reflect its high concentration in vivo when present.

**Fig. 7:**
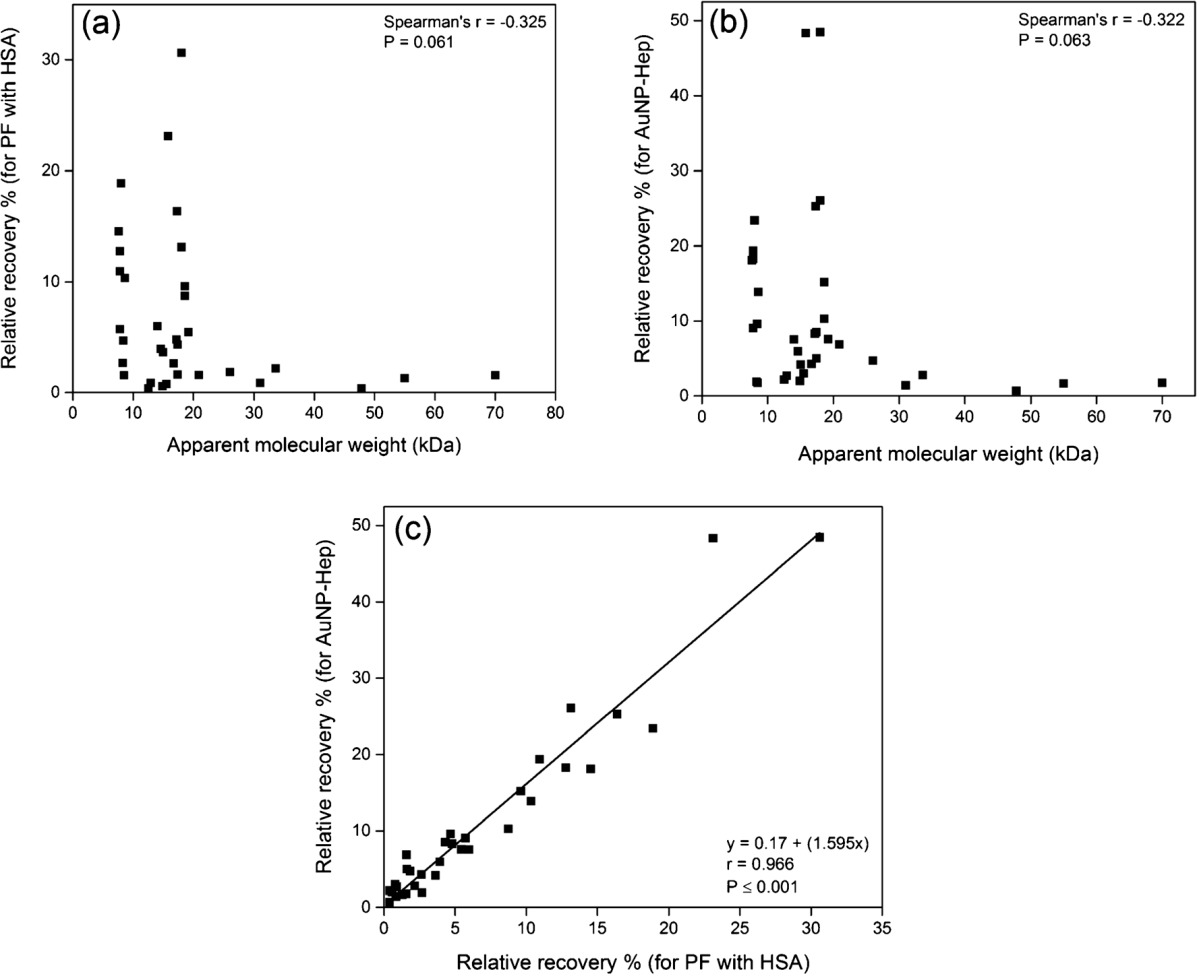
Relative recoveries (%) for the 34 cytokines versus apparent molecular weight, and a comparison between AuNP-Hep and perfusion fluid (*PF*) with HSA. **a** RR % using PF with HSA versus apparent molecular weight (kDa). **b** RR % using AuNP-Hep versus apparent molecular weight (kDa). The original RR (%) data can be found in the ESM (Table S4). Spearman *r* and *p* values are shown in each scatterplot; the *p* values were not significant in both **a** and **b**. **c** RR % for PF with HSA versus RR % with AuNP-Hep; the slope for the linear regression is 1.595 (*p* ≤ 0.001)

Further comparison showed non-linear inverse trends between the RR (%) and the apparent molecular weight for the 34 cytokines, when sampled using the PF with HSA (Fig. 7a) and the AuNP-Hep (Fig. 7b). These inverse trends were statistically similar (Spearman’s *r* = −0.325 and −0.322, *p* = 0.061 and 0.063, respectively). A plot of RR using AuNP-Hep (*y*-axis) versus RR with PF using HSA (*x*-axis) gave a statistically significant positive correlation (linear regression, *r* = 0.966, *p* ≤ 0.001) (Fig. 7c). The gradient of the line fitted was 1.595; therefore, an overall factor of 1.595 improvement in RR was afforded by the AuNP-Hep relative to that achieved with PF with HSA.

**Fig. 8:**
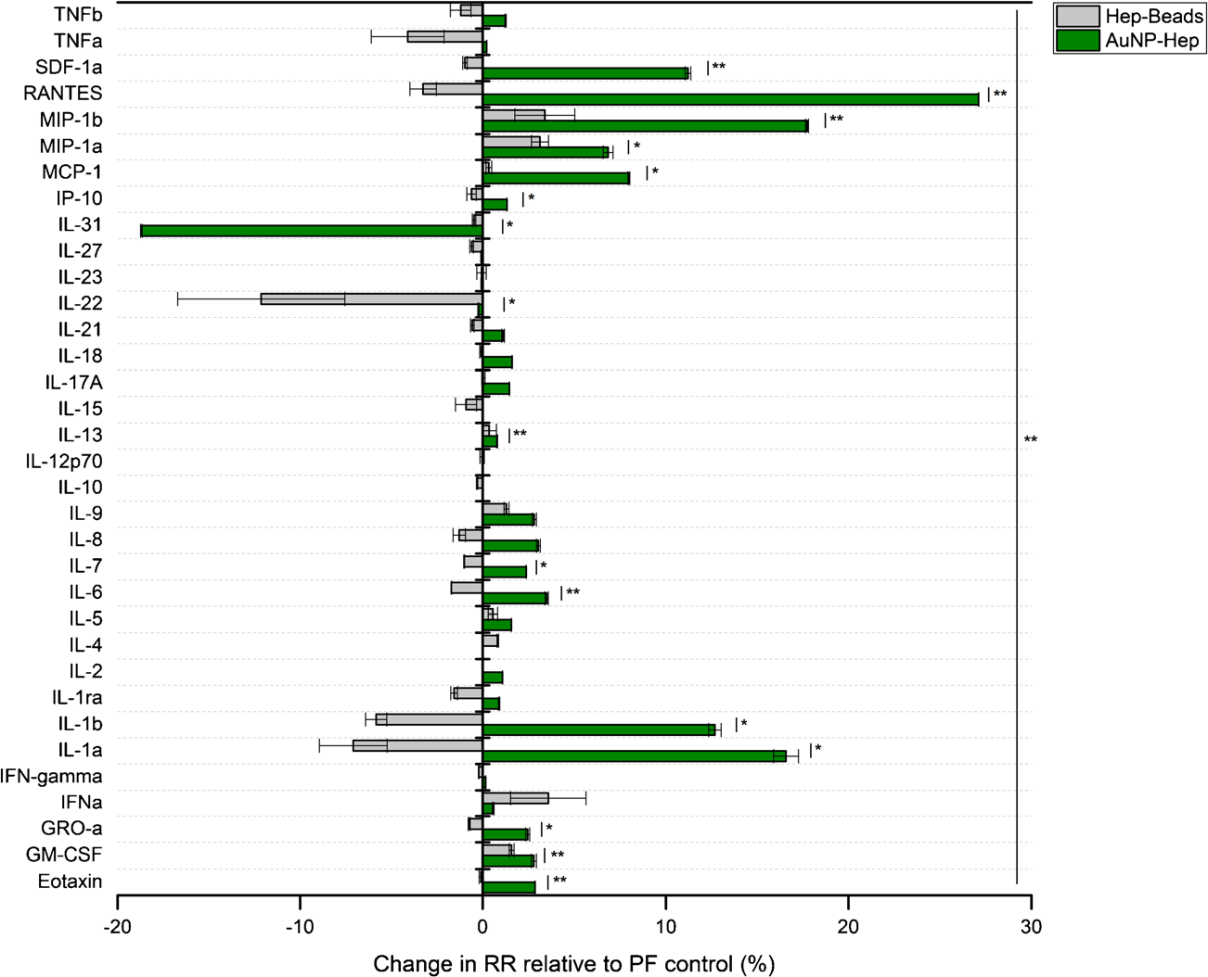
The change in relative recovery (RR %) of 34 human cytokines relative to a PF control, sampled by microdialysis in vitro; a comparison between AuNP-Hep and Hep-beads. The average RR (%) relative to the PF with HSA control was calculated from 48 h worth of sampling (at 12 h sample intervals), from at least two repeat experiments analysed in duplicate. *Error bars* denote SEM. Significant differences were observed between individual cytokines sampled using AuNP-Hep versus Hep-beads (paired sample t test) and between the overall concentrations obtained using the two sampling methods (one-way ANOVA). Statistical significance is denoted as follows: *p ≤ 0.05 and **p ≤ 0.01

Based on the improved cytokine recovery achieved by the AuNP-Hep compared to the PF control, the performance of the AuNP-Hep was then tested in vitro and compared with the Hep-beads developed by Duo and Stenken [8, 9]. These Hep-beads were developed using commercial amine-functionalised polymeric microspheres (ca. 6.0 μm), with heparin bound directly to the functionalised surface ligand [9]. In vitro studies performed by Duo and Stenken showed that Hep-beads performed well in terms of binding specificity and stability [9]. In vivo sampling of monocyte chemoattractant protein-1 (or CCL2) was performed using microdialysis probes implanted into the peritoneal cavity of rats. The test catheters were perfused with fluid containing either Hep-beads or free heparin at a flow rate of 1.0 μL/min [8]. In the present study, the Hep-beads were prepared using a modified version of the method described by Duo and Stenken, and in vitro microdialysis sampling was performed alongside a PF control in the same way as for the AuNP-Hep [8, 9]. It was hypothesised that the AuNP-Hep would yield a similar RR to that of the Hep-beads in vitro. A comparison between the average RR (%) achieved using the AuNP-Hep versus the Hep-beads, relative to the PF control, is shown in Fig. 8. In vitro sampling performed using the AuNP-Hep resulted in significantly improved RR over a PF control when compared with the Hep-beads (*p* ≤ 0.01; one-way ANOVA; significance for individual cytokines is as shown in Fig. 8). The use of the AuNP-Hep improved recovery for all but one of the cytokines sampled for, namely IL-31, which showed a decreased RR in line with the results of previous experiments (as shown in Fig. 6). Fifteen of the cytokines sampled for by AuNP-Hep had significantly improved RR when compared with the Hep-beads (*p* ≤ 0.05 or 0.01; paired sample *t* test). These results clearly show the efficacy of the AuNP-Hep for enhancing cytokine recovery during microdialysis in vitro, in a setting representative of clinical monitoring.

One of the problems associated with using Hep-beads for enhanced microdialysis sampling is that the beads are prone to settling. This was identified by Duo and Stenken, who used a custom-made rotating shaker to keep the syringe pump in motion during microdialysis, as well as varying the flow rate between 0.5 and 1 μL/min (and higher) to encourage bead movement [8]. During brain microdialysis performed in a clinical setting, the catheter itself is secured during surgery, and tape is sometimes used to hold the tubing in place on the ward. Nevertheless, it is imperative to move the microdialysis system as little as practically possible, to minimise risk to both the fragile probe and the patient. The microdialysis pump and collection vial are situated immediately beside the patient’s head, generally resting on the pillow or bedding. In the clinical setting, it is clearly not practical to use a rotating shaker, as this would disturb the microdialysis setup and pose a general hazard to critical care. Furthermore, microdialysis in our NCCU is performed using pumps with a fixed flow rate of 0.3 μL/min, so using a variable flow rate is not possible in this context. In this in vitro study, a small 3D oscillating platform was employed in an attempt to emulate the movement achieved by the custom rotating shaker used by Duo and Stenken [8]. Even with this in place, we observed that the beads had settled at the beginning of the 48-h sampling period. The perfusion rate of the beads was significantly reduced from the original concentration in the syringe, as shown in Table 3. Our average bead recovery of ca. 3% was significantly lower than the ca. 93% achieved by Duo and Stenken, likely due to the decreased flow rate and different probe types used in this study [8].

**Table 3:** Hep-bead concentrations (beads/mL) before, during and after in vitro microdialysis. Values are mean ± SD of three separate experiments, based on 10 readings of each bead amount using a haemocytometer

	0 h (before)	Syringe; microdialysate; 24 h	Microdialysate; 48 h	Syringe; 48 h (after)
PF2^a^	42.8 ± 4.9 × 10^5^	1.0 ± 0.4 × 10^5^	1.0 ± 0.4 × 10^5^	51.5 ± 2.7 × 10^5^
PF3^a^	45.4 ± 3.5 × 10^5^	1.9 ± 0.5 × 10^5^	1.9 ± 0.7 × 10^5^	45.6 ± 3.5 × 10^5^

^a^PF2 comprises Hep-beads in PF with 35 mg/mL HSA; PF3 comprises Hep-beads in PF with 1 mg/mL HSA

**Fig. 9:**
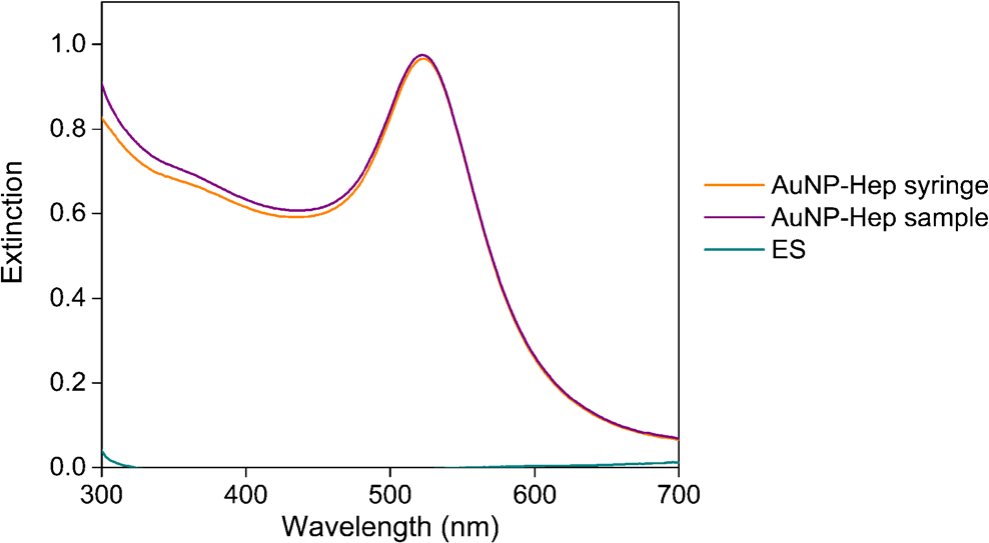
Typical UV-Vis absorption spectra of the PF containing AuNP-Hep before (from the syringe) and after (in the sample prior to decoupling) in vitro microdialysis sampling, as well as the spectrum of the external solution (*ES*) after microdialysis. The concentration of the AuNP-Hep is reflected by the peak extinction at ca. 520 nm (AuNP-Hep syringe = 4.3 nM, AuNP-Hep sample = 4.4 nM and ES = 0 nM)

The poor perfusion and recovery of the Hep-beads in our in vitro setting representative of clinical microdialysis emphasises the need for an alternative approach capable of remaining suspended during sampling. Based on visual observations, the AuNP-Hep used in this study appeared to remain in solution during sampling based on the characteristic burgundy colour of the PF in the syringe, tubing and sample vial. These observations were confirmed by UV-Vis analysis of the PF containing AuNP-Hep before (from the syringe) and after microdialysis of the samples (in the samples prior to decoupling). The concentration of the AuNP-Hep was marginally higher (approximately 2%) in the sample than what was originally in the syringe, as shown by the slight increase in peak extinction at ca. 520 nm (Fig. 9). This result confirmed that the perfusion and recovery of the AuNP-Hep during microdialysis was improved compared to the Hep-beads. The slight increase in AuNP-Hep in the sample is thought to be due to the slight evaporation of fluid from the collection vial during the 12-h sampling periods.

Safety is a serious concern for the potential clinical utility of microdialysis catheters with perfusion fluids containing heparin and AuNP. Heparin is an anticoagulant with known effects in the human body. Although AuNP are considered relatively inert, their physiological behaviour remains poorly characterised [23]. As such, both heparin and AuNP should be used with caution in a clinical setting. In the context of this work, it is therefore imperative to demonstrate that the AuNP-Hep are retained within the catheter during in vitro microdialysis sampling. Wang et al. have previously shown, during in vitro microdialysis using PF supplemented with 0.1 μM heparin, that the heparin passes through the microdialysis catheter in only very small amounts (2.7 ± 0.9%, *n* = 3) [6]. When in vitro sampling was performed using Hep-beads, Duo and Stenken reported that no heparin could be detected in the ES [8]. In this study, we tested for heparin and AuNP in the ES by Azure A assay and UV-Vis, respectively. Neither heparin (results not shown) nor AuNP (Fig. 9) were detected in the ES using the aforementioned detection methods. During one of the AuNP-Hep in vitro sampling experiments, AuNP-PEG were applied as a negative control (as the AuNP-PEG are stable under physiological conditions but do not contain heparin) in one of the three catheters used. The AuNP-PEG passed through the catheter during this experiment, resulting in a subtle visible colour change in the ES, and a small AuNP extinction peak at approximately 520 nm when measured by UV-Vis (results not shown). The slight peak in absorption reflected an AuNP concentration of 0.07 nM in the ES when sampling was performed using an AuNP-PEG control. When the in vitro sampling was repeated using two catheters perfused with AuNP-Hep and PF control, respectively, no AuNP were visible in the ES. UV-Vis analysis of this ES also did not yield a peak at ca. 520 nm, and the concentration of AuNP in the ES was estimated at 0 nM (Fig. 9). These results suggest that, while the AuNP-PEG could pass through the catheter, the AuNP-Hep could not do so at the concentration levels detectable by UV-Vis. Although further analysis will be required to fully characterise the behaviour of the AuNP-Hep in the microdialysis catheter, these preliminary results suggest that the AuNP-Hep are retained within the catheter during in vitro sampling. Based on these results, enhanced microdialysis sampling of cytokines using AuNP-Hep is therefore a viable option for use in TBI patients.

## Conclusions

Microdialysis sampling of proteins can be challenging due to factors including their size and steric properties. Improving recovery via enhanced microdialysis increases the chance of detecting important extracellular changes in protein concentration, in environments such as the brain. In this study, we have shown that microdialysis sampling using AuNP-Hep improved the recovery of 34 human cytokines in an in vitro setting designed to represent clinical monitoring in NCCU patients. This enhanced recovery, yielding an increase in the final concentrations detected, could improve detection of cytokines in microdialysates, particularly those at low concentrations. The use of the AuNP-Hep would provide a suitable means of overcoming the practical constraints of applying the Hep-beads in a clinical setting, namely avoiding the need to rotate the pump during sampling to avoid the beads from settling. In addition, the AuNP-Hep provide a means of enhancing cytokine recovery without using blood products such as HSA that have been successfully used in the past [2, 5, 7], but have now become difficult to obtain in preparations suitable for clinical microdialysis. Increasing licencing restrictions on pharmacy manufacturing facilities mean that formulation with this blood product is either not allowed or else prohibitively expensive. This presents a significant logistical barrier to microdialysis sampling of proteins. Synthetic alternatives, such as AuNP-Hep, are therefore a feasible means of overcoming this barrier.

## Electronic supplementary material

ESM 1 (PDF 150 kb).
