# Supplementary material for: Heparin-gold nanoparticles for enhanced microdialysis sampling
Source: Anal Bioanal Chem. 2017 Jun 29;409(21):5031–42. doi: 10.1007/s00216-017-0447-y (PMC5534206; doi:10.1007/s00216-017-0447-y)
Supplement: Supplementary file 1 — (PDF 150 kb). [file 216_2017_447_MOESM1_ESM.pdf]

Analytical and Bioanalytical Chemistry

Electronic Supplementary Material

Heparin-gold nanoparticles for enhanced microdialysis sampling

Susan Giorgi-Coll, Holly Blunt-Foley, Peter J. Hutchinson, Keri L.H. Carpenter

Table S1 Cytokine binding test results; A comparison the relative recovery (RR; %) for the AuNP-PEG control versus the Heparin-AuNP. Average recovery, relative to the concentrations in the original sampling solution from three repeat experiments analysed in duplicate, is shown for all cytokines

Cytokine/Chemokine	AuNP-PEG control RR (%)	AuNP-Heparin RR(%)
Eotaxin	2.67	1.92
GM-CSF	3.93	5.94
GRO-alpha	5.73	9.07
IFN-alpha	5.45	7.59
IFN-gamma	2.63	4.28
IL-1alpha	30.61	48.44
IL-1beta	16.37	25.26
IL-1ra	4.78	8.34
IL-2	0.78	3.01
IL-4	0.58	1.99
IL-5	1.84	4.74
IL-6	1.59	6.87
IL-7	1.62	5.00
IL-8	4.68	9.60
IL-9	5.99	7.57
IL-10	8.75	10.28
IL-12p70	1.55	1.76
IL-13	0.38	2.19
IL-15	0.88	2.70
IL-17alpha	0.85	1.39
IL-18	13.14	26.08
IL-21	3.62	4.17
IL-22	2.16	2.80
IL-23	1.29	1.67
IL-27	0.38	0.68
IL-31	23.11	48.32
IP-10	1.56	1.77
MCP-1	10.34	13.90
MIP-1alpha	10.95	19.39
MIP-1beta	14.53	18.12
RANTES	12.78	18.27
SDF-1alpha	18.89	23.39
TNF-alpha	4.32	8.54
TNF-beta	9.62	15.19

Table S2 Results of the cytokine binding test, wherein the AuNP-Hep were incubated in a mixed standard solution of the 34 human cytokines. Unbound cytokines were then removed by washing, followed by decoupling of bound cytokines and detection. The concentration of the cytokines bound to the AuNP-Hep, versus the concentration bound to the AuNP-PEG negative control, are shown. The data represents the average of three repeat experiments, performed and analysed in duplicate (± S.E.M.). Cytokines with concentrations of < 5 pg/mL are listed here; the other cytokine concentrations are shown in Fig 5

Cytokine/Chemokine	AuNP-PEG control (pg/mL ± S.E.M.)	AuNP-Heparin (pg/mL ± S.E.M.)
Eotaxin	0.67 ± 0.47	0.48 ± 0.23
IFN-alpha	1.23 ± 0.09	1.71 ± 0.24
IL-7	0.32 ± 0.23	1.00 ± 0.41
IL-12p70	4.07 ± 0.55	4.60 ± 0.72
IL-15	1.09 ± 0.77	3.37 ± 2.06
IL-17alpha	0.63 ± 0.45	1.03 ± 0.45
IP-10	1.37 ± 0.66	1.55 ± 0.40

Table S3 The concentrations of 34 human cytokines from *in vitro* microdialysis sampling using AuNP-Hep. Average concentrations from 48 h worth of sampling (at 12 h sample intervals), from two repeat experiments analysed in duplicate (± S.E.M.), are shown. Cytokines with concentrations of < 5 pg/mL are listed here; the other cytokine concentrations are shown in Fig 6

Cytokine/Chemokine	AuNP-PEG control (pg/mL ± S.E.M.)	AuNP-Heparin (pg/mL ± S.E.M.)
Eotaxin	0.18 ± 0.08	0.90 ± 0.08
IFN-alpha	1.28 ± 0.18	1.40 ± 0.22
IL-7	0.15 ± 0.02	0.63 ± 0.02
IL-12p70	2.38 ± 0.27	2.38 ± 0.39
IL-15	ND	ND
IL-17alpha	0.63 ± 0.17	1.70 ± 0.14
IP-10	1.64 ± 0.23	2.80 ± 0.25

Table S4 The relative recoveries (%) of the 34 human cytokines sampled using *in vitro* microdialysis sampling; a comparison between the PF control and the AuNP-Hep. Average recovery, relative to the concentrations in the original sampling solution, from 48 h worth of sampling (at 12 h sample intervals), from two repeat experiments analysed in duplicate, are shown

Cytokine/Chemokine	PF control RR (%)	AuNP-Heparin RR(%)
Eotaxin	0.71	3.57
GM-CSF	5.57	8.36
GRO-alpha	10.30	12.76
IFN-alpha	5.67	6.24
IFN-gamma	1.22	1.37
IL-1alpha	36.89	53.48
IL-1beta	20.64	33.32
IL-1ra	4.22	5.11
IL-2	1.21	2.29
IL-4	1.58	1.43
IL-5	0.32	1.88
IL-6	4.03	7.52
IL-7	0.76	3.13
IL-8	8.52	11.55
IL-9	8.04	10.86
IL-10	0.76	0.76
IL-12p70	0.91	0.91
IL-13	1.34	2.12
IL-15	ND	ND
IL-17alpha	0.85	2.30
IL-18	3.64	5.24
IL-21	6.39	7.50
IL-22	2.47	2.23
IL-23	1.55	1.47
IL-27	0.28	0.19
IL-31	86.21	79.52
IP-10	1.86	3.18
MCP-1	25.62	33.59
MIP-1alpha	26.21	33.06
MIP-1beta	16.55	34.30
RANTES	35.02	62.14
SDF-1alpha	27.65	38.86
TNF-alpha	0.45	0.66
TNF-beta	4.45	5.69
